# Commencement

**Published:** 1869-04

**Authors:** 


### COMMENCEMENT.

The regular Annual Commencement Exercises, terminating the Twenty-third annual session of the Ohio College of Dental Surgery, were held on Wednesday evening, March 3d, in the first lecture room of the College. A large audience, consisting of members of the profession, friends of the Institution and others were present.

The exercises were commenced with prayer and reading the scriptures, by the Rev. H. D. Moore, after which an interesting address was delivered and the degrees conferred by Dr. G. W. Keely, President of the Board of Trustees. After this, an address on behalf of the Faculty, that called forth warm approval was delivered by Prof. E. Rives, to which a response was made on behalf of the senior class by C. W. Stephenson.

The senior class numbered nine members, each of whom received the honors of the college, whose names we give in this connection, viz.:

James Gamble, Indiana; J. L. Oldham, Ohio; T. O. Paine, Miss.; J. S. Cassidy, Ky.; J. F. Dennis, Ohio; C. W. Barnes, Wis.; C. W. Stephenson, Ohio; J. DeHart, Indiana; J. Kapp, Michigan.

The examinations of both Senior and Junior classes were eminently satisfactory, of this more particular mention may be made hereafter. The plan of dividing the studies into two courses, and the establishment of two classes is more satisfactory the longer it is tested—alike satisfactory to the students and the teachers. So entirely successful has this method been, where tried, and so fully verified and established by the experience of other institutions, that it is a matter of some surprise that Medical and Dental schools generally do not adopt the plan. The course of instruction in the Medical and Dental schools of our country lacks system and thoroughness; the course of study is too short—effort is made to teach altogether too much in the prescribed time. It would be a matter of great gratification to see a reform in this respect. T.

